# Overactive Bladder (OAB) Symptoms in Patients Undergoing Flexible Cystoscopy

**DOI:** 10.7759/cureus.63289

**Published:** 2024-06-27

**Authors:** Rafiullah Khan, Syed M Nazim, Shahid Iqbal

**Affiliations:** 1 Surgery, Aga Khan University Hospital, Karachi, PAK; 2 Medicine, Medical College, Aga Khan University, Karachi, PAK

**Keywords:** oab-v8 score, luts, flexible cystoscopy, oab, overactive bladder

## Abstract

Objective: The objective is to measure the change in overactive bladder (OAB) symptoms in patients undergoing flexible cystoscopy in the early postoperative period using a validated OAB-V8 tool.

Patients and methods: It was a prospective, cross-sectional, observational study conducted by a section of Urology at the Aga Khan University Hospital, Karachi. The total duration of the study was 12 months (July 2022 to June 2023). All adult patients who underwent flexible cystoscopy under local anesthesia for diagnostic and surveillance purposes were included in the study. OAB symptoms were evaluated using the validated eight-item OAB-V8 tool just before the cystoscopy and on postoperative days 1 and 4. Patients were categorized as either OAB-negative (<8) or OAB-positive (≥8) based on their sum scores. Mean sum scores of different variables and OAB subdomains were assessed.

Results: Sixty-three patients were included in the final analysis with a predominantly male population. The mean pre-cystoscopy (screen) score was 7.46 + 5.58, which increased to 9.89 + 6.82 on day 1 (p<0.01) before decreasing back to 7.68 + 5.7 on day 4 (p=0.08). Twenty-one patients (33.3%) were labeled OAB positive on day 0. Following cystoscopy, this number increased to 32 patients (50.8%) as 11 patients (26.2 %) developed de-novo OAB symptoms. The sub-group analysis showed an insignificant impact of age (p=0.5), gender (p=0.51), indication (p=0.22), and use of alpha-blocker (p= 0.30) on change in OAB score.

Conclusion: OAB symptoms are frequently encountered in patients undergoing awake (flexible) cystoscopy. This procedure can also trigger de novo OAB symptoms, albeit transiently, which typically resolve over time. This information could help in patient counseling, management, and the need for intervention in the future.

## Introduction

Cystoscopy stands as a pivotal endo-urological procedure and diagnostic tool for examining the urinary bladder via the urethra [1]. Studies indicate that both rigid and flexible cystoscopies are well-tolerated and safe for patients. However, flexible cystoscopy tends to induce less discomfort in comparison, regardless of gender [2,3]. Patients may experience symptoms such as urgency, frequency, dysuria, hematuria, and occasional urinary incontinence post-cystoscopy [4].

Defined by the International Continence Society (ICS), overactive bladder (OAB) encompasses symptoms like urinary urgency, with or without urgency incontinence, often coupled with frequency and nocturia [5]. Various patient-reported outcome questionnaires have been introduced to screen for OAB and assess symptom severity, including the Bladder Assessment Tool (BAT), Overactive Bladder Symptom Score (OABSS), and Overactive Bladder Questionnaire (OAB-V8) [6]. The OAB-V8 is a patient-reported outcome questionnaire that measures the degree of bother from four symptoms related to OAB, i.e., urinary frequency, urgency, nocturia, and urge incontinence on a 6-point Likert scale ranging from 0 (not at all) to 5 (a very great deal) with a maximum possible score of 40 [7]. Malde et al. tested different validated tools for assessing the OAB and found OAB-V8 to demonstrate the highest sensitivity (98%) [6].

Recently, Saratlija et al. observed the impact of both rigid and flexible cystoscopies on OAB symptoms. They found that cystoscopy can induce OAB symptoms in patients without a prior OAB history (de novo OAB), while patients with pre-existing OAB symptoms (OAB-positive) often experience a reduction in symptoms post-cystoscopy [8].

There is a lack of data regarding the prevalence of OAB in the urological population particularly regarding the relationship between OAB symptoms and patients undergoing flexible (awake) cystoscopy. Understanding this relationship could aid in identifying patients at risk, managing new symptoms, and providing counseling. Hence, we conducted this study aiming to measure changes in OAB symptoms post-flexible cystoscopy, utilizing the validated OAB-V8 tool at early post-operative stages (day 1 and day 4). In addition, we sought to explore potential factors such as gender, age, and suspected pathology/prior diseases that may influence changes in OAB symptoms following flexible cystoscopy as our secondary objective.

## Materials and methods

It was a single-center, prospective observational study carried out by the section of urology at the Aga Khan University Hospital. The study protocol was approved by the Institutional Ethical Review Committee (ERC ref: 2022-7365-21844). All patients undergoing flexible cystoscopy, meeting the inclusion criteria, during the one-year study period (July 2022 to June 2023) at our center were included in the study.

We employed a non-probability consecutive sampling method to prospectively enroll adult patients (> 18 years) presenting at the urology clinic, scheduled for flexible cystoscopy under local anesthesia for diagnostic or surveillance purposes between July 2022 and June 2023, who could comprehend simple English language. We excluded patients with active urinary tract infections, pregnant individuals, those with indwelling catheters, genitourinary (GU) tuberculosis, interstitial cystitis, or those planned for interventions such as cold cup biopsy/fulguration or stent removal. Patients already taking anticholinergic and/or B3-agonist medications or experiencing cognitive impairment and inability to communicate with staff were also excluded.

A comprehensive assessment including history, physical examination, urinalysis, culture/sensitivity, diagnostic ultrasonography of the bladder, and frequency volume charting was conducted initially. Written informed consent was obtained after explaining the procedure, study protocol, potential benefits, and risks, and the OAB-V8 screening tool to the patient.

Flexible cystoscopy was performed using an OlympusTM (Aizu Olympus Co., Ltd, Fukushima, Japan) size 15.3 Fr flexible cystoscope under local anesthesia. Standard antibiotic prophylaxis with Inj. Gentamycin 80 mg I/V or an alternative antibiotic (in case of allergy/contraindication) was administered. Local anesthetic gel Xylocaine 2% was instilled in the urethra, and male patients were positioned supine while female patients were positioned in the frog-leg supine position. Postoperative analgesia comprised Tab. Paracetamol two tablets up to three times per day for three to five days and a urinary tract alkalizer.

OAB symptoms were assessed using the validated eight-item OAB Screening Awareness Tool (OAB-V8) just before flexible cystoscopy, and two forms were provided to each patient in an envelope for completion on postoperative days 1 and 4. Questionnaires were collected during the follow-up visit after one week, and data were entered using a pre-designed Performa. Patients with significant symptoms were counseled on treatment strategies such as lifestyle modifications, bladder training, and appropriate medication(s).

Data were analyzed using SPSS v. 21 (IBM Corp., Armonk, NY). Patients were categorized into baseline positive, or negative OAB symptoms groups based on pre-cystoscopy screening (<8 and ≥8 sum-score on OAB-V8 tool, respectively). Mean ± SD/median (IQR) was used for descriptive statistics for quantitative variables, while frequency and percentages were used for qualitative variables. Differences between pre-cystoscopy and post-cystoscopy day 1 and day 4 scores were assessed using repeated measure ANOVA/Friedman test. Mann-Whitney U test/independent T-test determined differences in quantitative variables between OAB positive and negative groups. The chi-square test/Fisher exact test determined the relationship of qualitative variables with OAB status (positive or negative). The statistical significance level was kept at p<0.05.

## Results

During the study period, a total of 95 flexible cystoscopies were conducted, with 75 patients meeting the inclusion and exclusion criteria and being enrolled in the study. The remaining 20 patients were excluded due to tumor recurrence (five), bladder biopsies (eight), DJ stent removal (three), and unable to comprehend simple English language (four). Of these 75 patients, 63 (84%) diligently completed all three OAB questionnaires (pre-procedure, day 1, and day 4), thus being included in the final analysis. Among them, there were 47 (74.6%) male and 16 (23.4%) female patients (Table 1).

**Table 1:** Demographics of the study population LUTS - lower urinary tract symptoms

	n	Percentage
Number of patients	63	100
Gender		
Male	47	74.6
Female	16	23.4
Avg. Age	62.7 (SD: 13.6)	
Age>50	53	84.1
Age<50	10	15.9
Indication		
LUTS	01	1.58
Hematuria	07	11.11
Surveillance	53	84.1
Others	02	3.17
Medications (Alpha Blockers)		
Yes	20	31.7
No	43	68.3
Pre-cystoscopy Score		
OAB-Positive	21	33.3
OAB-Negative	43	67.7

In the sample population, the average pre-cystoscopy OAB score was 7.46 + 5.58, which increased to 9.89 + 6.82 on day 1 (p<0.01) before decreasing back to 7.68 + 5.7 on day 4. Significant differences were observed between the average scores pre-cystoscopy and on day 1 (p<0.01), as well as between day 1 and day 4 OAB scores (p<0.01). However, the difference between pre-cystoscopy and day 4 scores was not significant (p=0.08). ANOVA results indicated a notable change in OAB symptoms following flexible cystoscopy (p<0.01). Furthermore, women exhibited a higher mean baseline OAB score of 8.06 + 7.16 compared to men's 7.25 + 5.01, a pattern consistent across individual questions as well.

Using the OAB-V8 questionnaire cut-off values (0-7: OAB-Negative, 8-40: OAB-Positive), 42 patients (66.7%) were categorized as OAB-negative based on their pre-cystoscopy scores, while 21 patients (33.3%) were OAB-positive. Following the procedure, the number of patients with a positive OAB score increased to 32 patients (50.8%) on the first post-procedure day, with OAB-negative patients accounting for 49.8% (31 patients) of the study population. Among the initially OAB-negative patients (n=42), 11 patients (26.2% of the OAB-negative group) developed de novo OAB-positive symptoms, significantly decreasing to 4.7% on post-procedure day 4 (p<0.01). Demographic data for this patient subgroup are presented in Table 2.

**Table 2:** Patients with de-novo OAB symptoms LUTS - lower urinary tract symptoms, OAB - overactive bladder

Patients with de-novo OAB symptoms	n=11
Age, mean (years)	59.9(SD:16.5)
Age > 50	08
Age < 50	03
Gender	--
Male	09
Female	02
Indication	--
LUTS	01
Surveillance	10
Mean OAB	--
Day-0	4.72 (+/-1.73)
Day-1	11.90 (+/-2.38)
Day-4	5.54 (+/-2.38)

Male and elderly patients appeared to be more prone to developing de novo OAB symptoms, although these tendencies did not reach statistical significance. The indication for cystoscopy, pre-cystoscopy OAB score, and the use of alpha-blockers showed no significant impact on the development of de novo OAB symptoms.

Among patients initially identified as OAB-positive during the pre-cystoscopy assessment, there was an observed increase in mean OAB score from 13.9 + 4.82 to 16.7 + 6.07 (p=0.01) on the first post-cystoscopy day, which then decreased to a mean of 14.1 + 5.03 on post-cystoscopy day 4 (p<0.01). However, comparing pre-cystoscopy and day 4 scores, the overall net change on day 4 was found to be insignificant (p=1.0). Subgroup analysis also revealed no significant impact of age (p=0.5), gender (p=0.51), indication (p=0.22), or use of alpha-blockers (p=0.30) on the change in OAB score. Table 3 displays the OAB-positive and negative groups, while the mean scores for the total population and evaluated subgroups are presented in Table 4.

**Table 3:** Patients with and without overactive bladder (OAB) symptoms pre- and post-cystoscopy

Time	OAB-negative, n (%)	OAB-positive, n (%)	Total	De-novo OAB +ve, n (%)
Pre-cystoscopy	42 (67.7)	21 (33.3)	63	--
Day-1	31 (49.2)	32 (50.8)	63	11 (26.2)
Day-4	40 (63.5)	23(36.5)	63	02 (3.2)

**Table 4:** Mean scores for each of the eight screening questions regarding overactive bladder (OAB-V8)

Population	Frequency during day	Uncomfortable urge during day	Sudden urge to urinate	Accidental loss of urine	Nighttime urination	Waking up at night to urinate	Uncontrollable urge	Urine loss associated with strong desire	Sum score
Total Sample (n=63)									
Day-0	1.17 (+/- 1.10)	0.87 (+/-0.94)	0.5873 (+/-0.83)	0.1111 (+/-0.36)	1.47 (+/-1.29)	1.09 (+/-1.32)	0.50 (+/-0.89)	0.09 (+/-0.34)	7.46 (+/-5.58)
Day-1	1.68 (+/-1.30)	1.36 (+/-1.18)	1.15 (+/-1.64)	0.23 (+/-0.49)	1.76 (+/-1.38)	1.38 (+/-1.36)	0.71 (+/-0.95)	0.11 (+/-0.36)	9.89 (+/-6.82)
Day-4	1.41 (+/-1.18) (p=0.001)	0.92 (+/-0.97) (p=0.001)	0.60 (+/-0.83) (p=0.007)	0.11 (+/-0.36) (p=0.004)	1.46 (+/-1.29) (p=0.001)	1.09 (+/-1.24) (p=0.001)	0.42 (+/-0.73) (p=0.002)	0.07 (+/-0.32) (p=0.226)	7.68 (+/-5.68) (p=0.001)
Men (n=47)									
Day-0	1.08 (+/-1.08)	0.76 (+/-0.86)	0.48 (+/-0.71)	0.04 (+/-0.20)	1.38 (+/-1.26)	1.00 (+/-1.28)	0.44 (+/-0.87)	0.06 (+/-0.24)	7.25 (+/-5.01)
Day-1	1.55 (+/-1.28)	1.23 (+/-1.14)	0.87 (+/-1.01)	0.12 (+/-0.33)	1.63 (+/-1.30)	1.29 (+/-1.28)	0.61 (+/-0.82)	0.04 (+/-0.20)	9.38 (+/-6.23)
Day-4	1.31 (+/-1.19)	0.76 (+/-0.86)	0.48 (+/-0.71)	0.04 (+/-0.20)	1.40 (+/-1.27)	1.00 (+/-1.17)	0.36 (+/-0.67)	0.04 (+/-0.20)	7.44 (+/-5.08)
Women (n=16)									
Day-0	1.43 (+/-1.15)	1.18 (+/-1.10)	0.87 (+/-1.08)	0.31 (+/-0.60)	1.75 (+/-1.39)	1.37 (+/-1.45)	0.68 (+/-0.94)	0.18 (+/-0.54)	8.06 (+/-7.16)
Day-1	2.06 (+/-1.34)	1.75 (+/-1.23)	2.00 (+/-2.65)	0.56 (+/-0.72)	2.12 (+/-1.58)	1.62 (+/-1.58)	1.00 (+/-1.26)	0.31 (+/-0.60)	11.00 (+/-8.45)
Day-4	1.68 (+/-1.13)	1.37 (+/-1.14)	0.93 (+/-1.06)	0.31 (+/-0.60)	1.62 (+/-1.36)	1.37 (+/-1.40)	0.62 (+/-0.88)	0.18 (+/-0.54)	8.37 (+/-7.31)
OAB-Negative (n =42)									
Day-0	0.64 (+/-0.53)	0.47 (+/-0.50)	0.26 (+/-0.44)	0.02 (+/-0.15)	0.76 (+/-0.69)	0.38 (+/-0.66)	0.04 (+/-0.21)	0.01 (+/-0.01)	4.21 (+/-1.86)
Day-1	1.11 (+/-0.94)	0.90 (+/-0.79)	0.83 (+/-1.76)	0.04 (+/-0.30)	1.07 (+/-0.89)	0.71 (+/-0.83)	0.26 (+/-0.49)	0.23 (+/-0.15)	6.33 (+/-3.96)
Day-4	0.83 (+/-0.65)	0.50 (+/-0.50)	0.23 (+/-0.43)	0.02 (+/-0.15)	0.76 (+/-0.72)	0.42 (+/-0.63)	0.04 (+/-0.21)	0.01 (+/-0.01)	4.45 (+/-2.10)
OAB-Positive (n=21)									
Day-0	2.23 (+/-1.17)	1.66 (+/-1.11)	1.23 (+/-1.04)	0.28 (+/-0.56)	2.90 (+/-0.99)	2.52 (+/-1.16)	1.42 (+/-1.02)	0.28 (+/-0.56)	13.95 (+/-4.82)
Day-1	2.80 (+/-1.20)	2.28 (+/-1.30)	1.80 (+/-1.16)	0.61 (+/-0.58)	3.14 (+/-1.15)	2.71 (+/-1.23)	1.61 (+/-1.02)	0.28 (+/-0.56)	16.71 (+/-6.07)
Day-4	2.57 (+/-1.16)	1.76 (+/-1.13)	1.33 (+/-0.96)	0.28 (+/-0.56)	2.85 (+/-1.01)	2.42 (+/-1.07)	1.19 (+/-0.81)	0.23 (+/-0.53)	14.14 (+/-5.03)

## Discussion

OAB symptoms (defined as the presence of urgency with or without urge incontinence, frequency, and nocturia in the absence of any pathology), are estimated to have an overall prevalence of 10%-17% in the adult population [9,10].

There is limited data on the prevalence of OAB in people with urological problems, which is anticipated to be higher but often goes undiagnosed [11]. This study aimed to examine the impact of flexible cystoscopy, age, gender, comorbidities, and indication for flexible cystoscopy on changes in OAB symptoms using a validated questionnaire (OAB-V8) in a sample population of 63 patients, yielding some noteworthy findings.

The OAB-V8, an eight-item score derived from a 33-item OAB questionnaire (OAB-q), is easy to fill with a low rate of unanswered questions and high internal consistency [6]. It has been validated in multiple languages including English, French, Arabic, Danish, German, Italian, Korean, Polish, Spanish, and Turkish.

In our study, the rate of OAB-positive patients was 33.3% of the study sample, with male and female prevalence of 29.7% and 43.7%, respectively, which is lower than that reported by Saratlija et al. (44%) [8]. Using the same threshold and questionnaire, this was much lower than the 75% prevalence rate reported by Cheung et al. in male urologic veterans [12].

Flexible cystoscopy led to an increase in the mean sum score of OAB symptoms in the study population, but there was no statistically significant difference in mean scores between day 0 and day 4, suggesting that flexible cystoscopy can exacerbate OAB symptoms temporarily, with patients returning to baseline scores over time. The occurrence of new-onset (de novo) OAB symptoms in 26.2% of the study population on day 1 after flexible cystoscopy indicates that the passage of the cystoscope may exacerbate symptoms, although this effect is short-lived.

Saratlija et al. also made an intriguing observation that patients positive for OAB symptoms experienced a short-term decrease in their symptom scores, which they attributed to the dilatation of the lower urinary tract [8]. Unlike Saratlija et al., we did not observe a mean decrease in OAB score post-cystoscopy; instead, there was an average rise in the mean score, almost returning to baseline by day 4, which is more understandable. We excluded patients taking anticholinergics or B-3 agonist medications, whereas Saratlija et al. [8] did not capture medication intake, which could potentially affect OAB symptoms following cystoscopy and influence their results. We did not find any impact of age, gender, comorbidities, or indication for cystoscopy on OAB symptoms post-flexible cystoscopy.

Strengths of our study include its prospective design, use of a validated OAB-screening tool, and assessment of a non-selected population, reflecting patients in daily practice. Limitations include a small sample size and single-center design. The results of this study will inform patient counseling, management, and the need for intervention in the future.

## Conclusions

OAB symptoms are common in patients undergoing flexible cystoscopy. Flexible cystoscopy can transiently trigger de novo OAB symptoms in some patients which typically resolve over time, however, uninfluenced by gender, age, and indication for flexible cystoscopy. Proper patient counseling, accordingly, can prevent unnecessary hospital visits.

## Appendices

**Table 5:** Performa for data collection

Performa			
Subject ID #	____		
Date of filling the Performa	--	Date of Cystoscopy	--
AGE	--	>50	<50
Gender	Male/Female		
Indication of Flexible Cystoscopy	Recurrent UTI Bothersome LUTS Hematuria (Microscopic/ Gross) Check (Surveillance) cystoscopy Other		
Medications:	Alpha blockers (Y/ N)		
OAB Sum Scores			
Baseline(pre-cystoscopy)	______	OAB Screened	-ve (<8) +ve(=>8)
Day-1	_______		
Day-4	______		
